# Brain fog with long covid and chemotherapy: systematic review and meta-analysis

**DOI:** 10.1136/bmjment-2025-301969

**Published:** 2025-12-17

**Authors:** Jack Christopher Wilson, Kathy Y Liu, Emma Mittelman, Polen Bareke, Eli Shleifer, Robert Howard

**Affiliations:** 1 Psychiatry, UCL, London, UK; 2 North London NHS Foundation Trust, London, UK; 3 UCL Medical School, London, UK

**Keywords:** Cognition Disorders, Mood Disorders, Neurocognitive Disorders

## Abstract

**Question:**

What are the cognitive, functional and affective characteristics of brain fog in individuals with long covid and following chemotherapy, and how are these features assessed across studies?

**Study selection and analysis:**

In March 2024, we conducted a systematic review and meta-analysis of peer-reviewed studies assessing cognition, function or mood in adults (≥18 years) with brain fog after COVID-19 or chemotherapy. PubMed, Embase and Web of Science were searched systematically according to eligibility criteria to March 2024, with an update in May 2025. Random-effects meta-analyses using the ‘dmetar’ package (V.0.0.9000) in R V.4.3.1 were performed for studies comparing individuals with and without brain fog. Bias was assessed using the National Institutes of Health Study Quality Assessment Tools.

**Findings:**

Of 3077 records screened, 65 studies met inclusion criteria: 40 investigated brain fog in long covid and 25 in chemotherapy populations. Considerable variation in assessment tools was observed. Montreal Cognitive Assessment was the most common cognitive test in long covid studies; Functional Assessment of Cancer Therapy—Cognitive Function was most used in chemotherapy studies. Nine long covid studies were eligible for meta-analysis. Compared with controls, individuals with brain fog had significantly lower cognitive performance (Hedge’s g=−0.63, 95% CI −1.15 to −0.12), higher fatigue (Hedge’s g=2.64, 95% CI 0.41 to 4.86) and more depressive symptoms (Hedge’s g=1.48, 95% CI 0.40 to 2.55). Heterogeneity was high (I^2^>70%). No chemotherapy studies were appropriate for meta-analysis, preventing direct comparison of brain fog features between long covid and chemotherapy groups.

**Conclusions:**

Brain fog in long covid and chemotherapy populations is associated with cognitive complaints, fatigue and mood disturbance, though assessment methods differ widely. To improve comparability and clinical understanding, we propose adoption of consistent tools and definitions in future studies. This will be a crucial step in generating findings that can be meaningfully compared across populations.

**PROSPERO registration number:**

CRD42024520549.

**WHAT IS ALREADY KNOWN ON THIS TOPIC**

- Brain fog is a subjective experience of reduced mental clarity, often including difficulties with memory and concentration. It is reported in several conditions, including long covid and following chemotherapy. Although multiple biological and psychosocial mechanisms have been proposed, there is no agreed definition, diagnostic approach or standardised way to measure brain fog, and the tools used to assess it vary widely across studies.

**WHAT THIS STUDY ADDS**

- This review and meta-analysis shows that researchers use a wide variety of tests to assess brain fog, with no consistent approach to definition or measurement. In long covid, brain fog is associated with small reductions in objective cognitive performance but much larger increases in fatigue and depressive symptoms. A comparable meta-analysis could not be performed for chemotherapy-related brain fog due to a lack of studies directly comparing affected and unaffected patients.

**HOW THIS STUDY MIGHT AFFECT RESEARCH, PRACTICE OR POLICY**

- The findings highlight the urgent need for a clear, shared definition of brain fog and standardised diagnostic and assessment tools. Adoption of common measures would enable more comparable studies, help clarify the mechanisms underlying brain fog and support the development and evaluation of targeted treatments.

## Introduction

Brain fog broadly refers to the subjective impression of reduced cognitive function, commonly associated with difficulties with concentration, mental clarity and memory, sometimes with accompanying symptoms, such as headache and mental fatigue.^1^ However, the imprecise, variable and subjective nature of brain fog has made it challenging to define and measure consistently. To date, there has been no systematic synthesis of the cognitive, functional and affective features most commonly reported in brain fog, nor of the tools used to assess them, particularly across long covid and chemotherapy populations. Our review was designed to address this gap.

Complaints of brain fog are common in patients with fibromyalgia and chronic fatigue syndrome.^2 3^ More recently, the concept has gained prominence in relation to patients with long covid, where it is frequently reported as a persistent symptom.^4^ A similar phenomenon has been widely documented among patients with cancer undergoing chemotherapy, often termed ‘chemobrain’, ‘chemofog’ or chemotherapy-related cognitive impairment (CRCI).^5^ A meta-analysis focusing on patients who received fluorouracil (5-FU) based chemotherapy reported an overall prevalence of cognitive impairment of 32%.^6^ Despite their occurrence in apparently widely different clinical populations, descriptions of brain fog in long covid, CRCI, fibromyalgia and chronic fatigue syndrome share clear phenomenological similarities, raising the question of whether a common underlying mechanism exists.^7 8^

Suggested pathophysiological processes underlying brain fog range from neuroinflammation, glial cell activation, blood–brain barrier dysfunction, hypoxaemia and hypometabolism in frontoparietal cortex, as well as effects of psychological factors, including mood disturbances and anxiety.^9^,^13^ In the long covid population, both viral persistence following infection and the generation of antibodies have been suggested as potential causes of persisting symptoms, though definitive evidence for each of these hypotheses is lacking.^14 15^ Brain fog may also represent a symptom of functional cognitive disorder, in which no identifiable biological abnormalities underlie the symptoms.^16^ Despite ongoing research, no single underlying mechanism has been convincingly identified.

Given this complexity, an essential first step in understanding brain fog would be to systematically characterise the associated cognitive, functional and affective features described in different clinical populations.^17^ However, this is challenging due to the variability in assessment tools used across studies. To address this, we aimed to systematically review the literature on the assessment of the features of brain fog in two distinct clinical populations: people with long covid and CRCI. Long covid has gained increasing clinical attention due to rising prevalence, while chemotherapy-related brain fog has traditionally been considered to have a biological basis related to the effects of anticancer medications on neuronal functioning.^18^ Through a review of published studies and examining standardised scale score differences, we aimed to identify the cognitive, functional and affective characteristics of brain fog in these two patient groups, thereby contributing to a clearer characterisation of this phenomenon. Our hope is that by characterising the features of brain fog and identifying the tools currently used to assess it, this review will contribute to the development of more consistent criteria and measurement approaches. In turn, such standardisation will enable more comparable studies, improve understanding of the phenomenon and ultimately support the identification of evidence-based interventions to reduce the morbidity experienced by the substantial proportion of patients affected.

## Methods

This systematic review adhered to a preregistered protocol (PROSPERO registration: CRD42024520549) and results are reported in accordance with the Preferred Reporting Items for Systematic Reviews and Meta-Analyses guidelines.

### Search strategy

We systematically searched PubMed, Web of Science and Embase up to 2 March 2024 without date restrictions. The following search syntax was used: (“Long covid” OR “Long-covid” OR “chemotherapy”) AND (“brain fog” OR “brain-fog” OR “brainfog” OR “chemobrain” OR “chemo brain” OR “chemo-brain” OR “chemofog” OR “chemo-fog” OR “chemo fog” OR “chemotherapy-induced cognitive impairment”) AND (cognit* OR “memory” OR function* OR “affective” OR “mood” OR depress* OR “anxiety”).

### Eligibility

We included peer-reviewed studies involving human participants aged 18 years or older reporting subjective or objective cognitive complaints, that is, brain fog, chemofog or chemotherapy-induced cognitive impairment, following chemotherapy or COVID-19 infection. Only studies with an exclusive cognitive impairment group identified as having ‘brain fog’ or a related term were included. Eligible study designs included observational studies (cohort, case–control, cross-sectional) and interventional studies (randomised controlled trials). We excluded abstracts, conference reports, letters, case reports and reviews. Studies involving participants with primary central nervious system (CNS) malignancies, CNS metastases or those addressing acute COVID-19 infection were excluded.

### Screening

Two out of four reviewers (JCW, PB, EM and ES) independently screened all titles and abstracts identified from the search. Articles meeting the inclusion criteria were selected for full-text review. Discrepancies were resolved through discussion and, if necessary, arbitrated by a third reviewer (JCW).

### Data extraction

Two out of four reviewers (JCW, PB, EM and ES) independently assessed the full text of eligible studies to confirm eligibility and extract relevant data. A standardised data extraction form was developed to capture: (1) general study information (author, year, title); (2) main outcome measure; (3) study design (sample size, single/comparative); (4) participant demographics (age, gender, ethnicity); (5) cognitive characteristics (assessment tools, mean score, SD, SE); and (6) functional and affective characteristics (assessment tools, mean score, SD, SE). Any uncertainties were resolved by an independent reviewer (JCW).

### Meta-analysis

Following a systematic review of the literature, we were able to perform meta-analyses to compare cognitive, functional or mood/ anxiety scale scores between brain fog (or equivalent) groups and either healthy controls or participants without brain fog who had long covid or chemotherapy.

Only studies that provided quantitative differences on cognitive, functional or mood/anxiety scales between brain fog versus no brain fog groups, including SD or SE measures, were included. Where studies used more than one cognitive, functional or mood or anxiety test, a hierarchy determined which was used, with global tests of cognitive, functioning or mood or anxiety, selected over more targeted testing. Where possible, tests shared widely between studies were also preferentially selected over tests specific to one study.

Suitable studies were included in a random effects meta-analysis model to compute pooled weighted effect sizes and 95% CIs across studies using the ‘dmetar’ package (V.0.0.9000) in R V.4.3.1. Effect sizes (Hedge’s G) were the between-group standardised mean differences (Cohen’s d) for cognitive, functional or mood or anxiety scores for brain fog versus no brain fog groups, represented as forest plots. Study heterogeneity was measured using the I^2^ statistic.

### Quality assessment

Methodological quality and risk of bias were assessed using the National Institutes of Health (NIH) Study Quality Assessment Tools.^19^ The checklists include criteria such as clarity of research question, definition of study population, participation rate, sample size justification, exposure and outcome measurement, blinding of assessors, adjustment for confounders and appropriateness of statistical analyses. Each item was rated as ‘yes’, ‘no’, ‘cannot determine’ or ‘not applicable’. Controlled intervention studies and observational cohort or cross-sectional studies were scored on a 14-point scale (0–4 low, 5–9 medium, 10–14 high quality), while case–control and before–after studies without a control group were scored on a 12-point scale (0–3 low, 4–8 medium, 9–12 high quality). Discrepancies were resolved by consensus, with arbitration by a third reviewer where necessary. The NIH National Heart, Lung, and Blood Institue website (https://www.nhlbi.nih.gov/health-topics/study-quality-assessment-tools) contains the full checklists.

## Results

### Study selection

**Figure 1:**
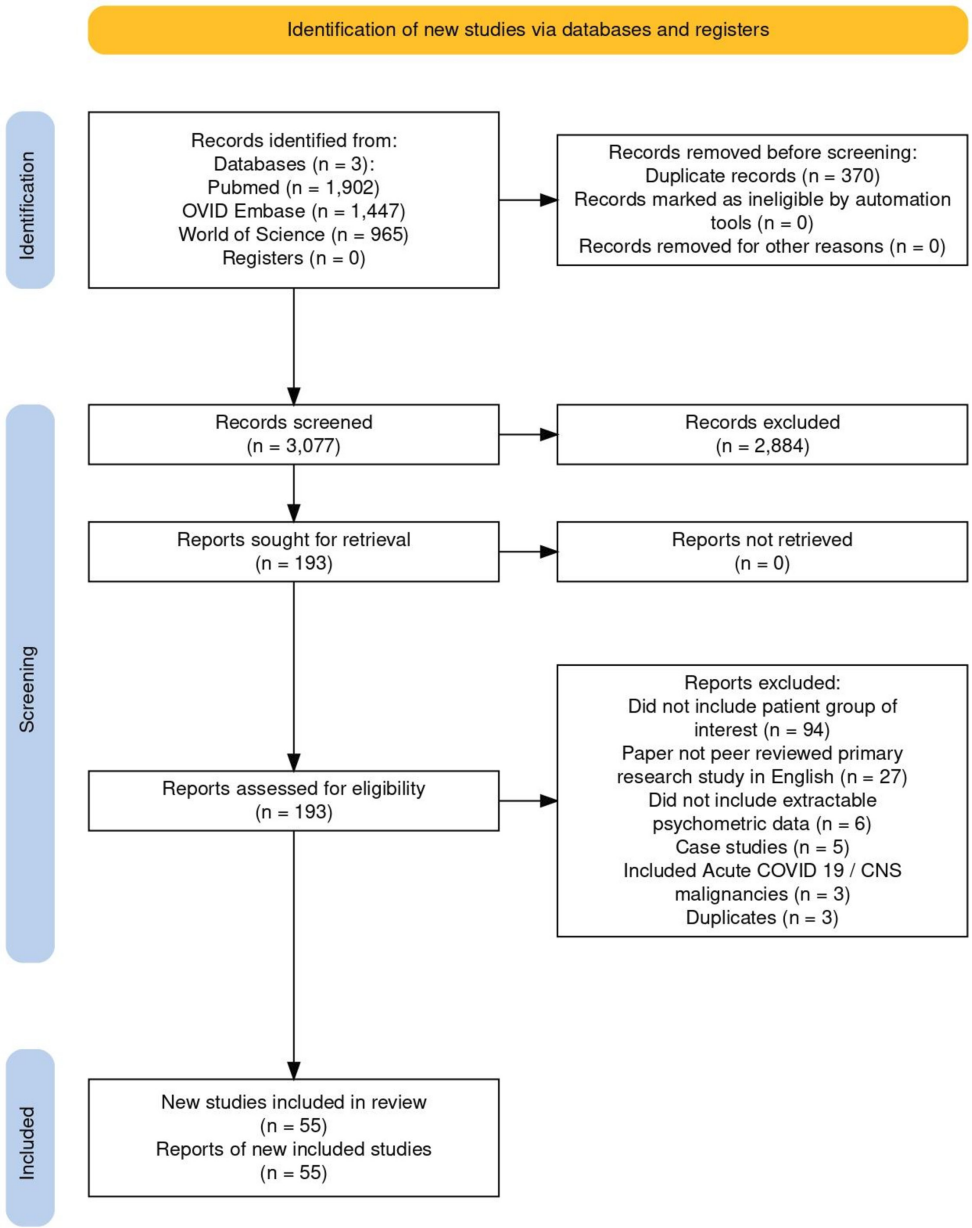
Preferred Reporting Items for Systematic Reviews and Meta-Analyses 2020 flow diagram for new systematic reviews, which included searches of databases and registers only. CNS, central nervous system.

The systematic search yielded 3077 unique studies, of which 55 met the inclusion criteria following abstract and full-text screening (figure 1). The final dataset included 31 studies focused on brain fog following COVID-19 and 24 in chemotherapy-related brain fog. The updated literature search conducted on 02 May 25 returned a further 344 abstracts, of which 10 met the inclusion criteria (9 COVID-19 brain fog-related studies and 1 chemotherapy-related brain fog study).

### Use of neuropsychological assessments

There was a large variety of cognitive, functional and mood/anxiety tests employed by researchers across different studies.

In the long covid studies, the most widely used cognitive test was the Montreal Cognitive Assessment (MoCA)^20^ (17 studies), followed by the Trail Making Test (TMT)^21^ (9 studies), Stroop^22^ (8 studies) and Digit Span^23^ (6 studies) tests. The most commonly employed functional tests assessed fatigue, with the Fatigue Severity Scale (FSS) being most widely used^24^ (11 studies), followed by the Chalder Fatigue Scale (CFS)^25^ (6 studies). In addition to fatigue scales, quality of life scales were widely employed, with the WHO Quality of Life—Brief^26^ and the EuroQol-5 Dimension^27^ each being employed by three studies. The most commonly used mood/anxiety assessments were measures of depression, with the Beck Depression Inventory—Second Edition (BDI-II)^28^ being employed by nine studies, the Hospital Anxiety and Depression Scale^29^ being employed by seven studies and the Patient Health Questionnaire-9 (PHQ-9)^30^ being employed by six studies. The most commonly used measure of anxiety was the Generalized Anxiety Disorder-7^31^ (five studies) (online supplemental table 1).

In chemotherapy studies, the most widely used assessment of cognition was the Functional Assessment of Cancer Therapy—Cognitive Function (FACT-Cog)^32^ (15 studies), a self-report questionnaire designed to assess subjective cognitive functioning in patients with cancer. Other cognitive tests employed included TMT^21^ (seven studies), Digit Span^23^ (seven studies) and the Rey Auditory Verbal Learning Test^33^ (five studies). Three studies employed the Mini-Mental State Examination^34^. A variety of functional tests were employed. Three studies included the Patient-Reported Outcomes Measurement Information System (PROMIS)^35^, a collection of self-report measures developed by the NIH, which includes a variety of functional measures, including fatigue, sleep disturbance, physical function, social participation and overall quality of life (in addition to submeasures of anxiety, depression and cognitive function). Three studies employed the Functional Assessment of Cancer Therapy—Fatigue^36^, and two studies employed the Functional Assessment of Chronic Illness Therapy (FACIT)^37^. The most commonly used measure of depression was the BDI-II ^28^ (four studies), followed by the PHQ-9^30^ (three studies). The most commonly used measure of anxiety was the State-Trait Anxiety Inventory^38^, used by three studies (online supplemental table 2).

### Study quality

The majority of chemotherapy studies were medium (17 studies) or high (7 studies) quality with 1 low-quality study (online supplemental table 3). The majority of long covid studies were medium (28 studies) or high (9 studies) quality, with 3 low-quality studies (online supplemental table 4).

### Meta-analyses of cognitive, functional and mood/anxiety scores

Nine long covid studies were eligible for meta-analysis. No chemotherapy studies compared patients who were experiencing brain fog with a group that did not report brain fog, which precluded an equivalent meta-analysis for chemotherapy patients.

**Figure 2:**
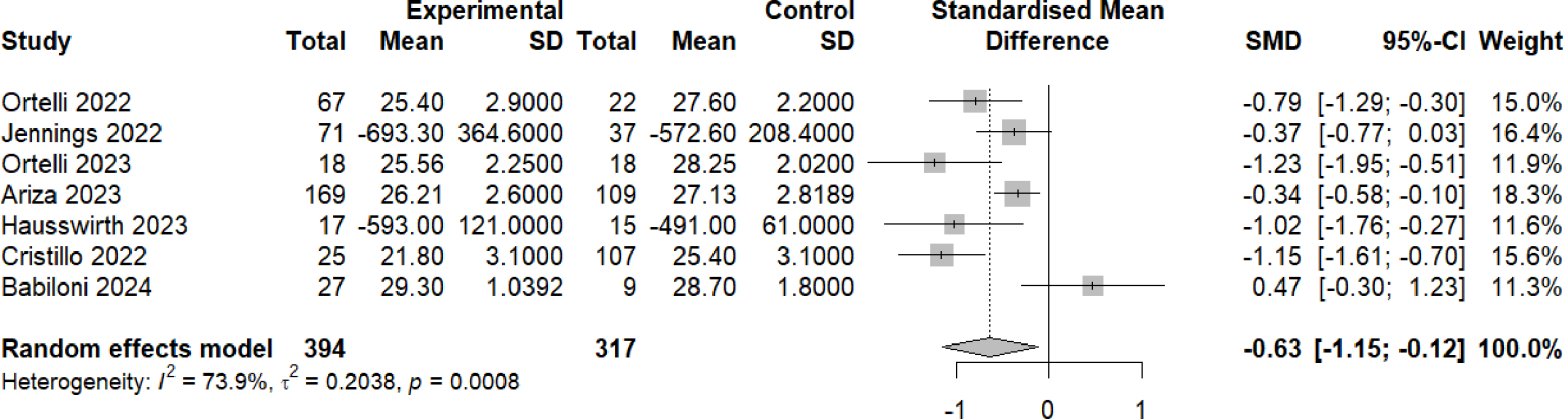
Cognitive scores post-COVID-19 in individuals with brain fog versus individuals without brain fog. Forest plot showing the effect size (Hedge’s G) and the pooled effect size for studies comparing individuals with brain fog post-COVID-19 (‘experimental’) versus individuals without brain fog (‘controls’). These were either health controls (Ortelli 2022,^61^ Ortelli 2023,^62^ Ariza 2023,^63^ Hausswirth 2023^64^) or individuals post-COVID-19 who did not have brain fog (Jennings 2022,^65^ Cristillo 2022,^66^ Babiloni 2024^67^). ‘Total’ denotes the sample size and SMD denotes standardised mean difference.

For the long covid studies, meta-analysis of cognitive test scores revealed a statistically significant difference between participants with brain fog and controls (Hedge’s g=−0.63, 95% CI (−1.15 to −0.12), p=0.0008) (figure 2). To put this effect size in a clinical context, this would be roughly equivalent to an MoCA score reduction of 1.44 points in participants with brain fog compared with control participants (calculated by multiplying the pooled SD of mean MoCA score for studies which used the MoCA (2.28) by the standardised mean difference across all studies (−0.63))

**Figure 3:**
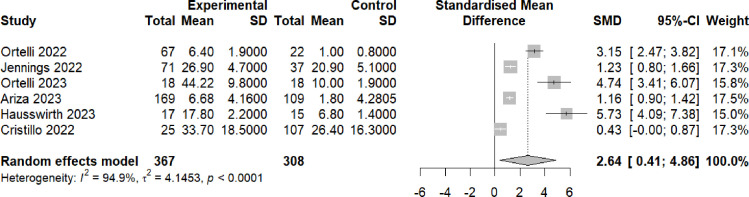
Fatigue scores post-COVID-19 in individuals with brain fog versus individuals without brain fog. Forest plot showing the effect size (Hedge’s G) and the pooled effect size for studies comparing individuals with brain fog post-COVID-19 (‘experimental’) versus individuals without brain fog (‘controls’). These were either health controls (Ortelli 2022,^61^ Ortelli 2023,^62^ Ariza 2023,^63^ Hausswirth 2023^64^) or individuals post-COVID-19 who did not have brain fog (Jennings 2022,^65^ Cristillo 2022.^66^) ‘Total’ denotes the sample size and SMD denotes standardised mean difference.

Six of the eight long covid studies that were eligible for meta-analysis employed measures of fatigue. Participants with brain fog demonstrated significantly higher levels of fatigue compared with participants without brain fog (Hedge’s g=2.64, 95% CI 0.41 to 4.86, p<0.0001) (figure 3).

**Figure 4:**
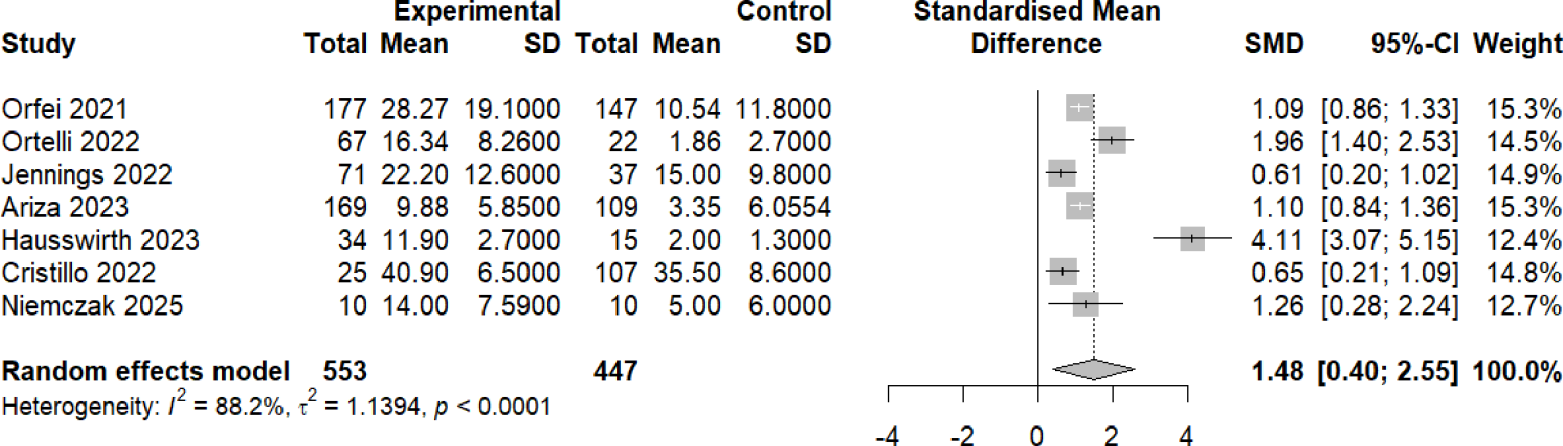
Mood scores post-COVID-19 in individuals with brain fog versus individuals without brain fog. Forest plot showing the effect size (Hedge’s G) and the pooled effect size for studies comparing individuals with brain fog post-COVID-19 (‘experimental’) versus individuals without brain fog (‘controls’) These were either health controls (Orfei 2021,^13^ Ortelli 2022,^61^ Ariza 2023,^63^ Hausswirth 2023,^64^ Niemczak 2025^68^) or individuals post-COVID-19 who did not have brain fog (Jennings 2022,^65^ Cristillo 2022.^66^) ‘Total’ denotes the sample size and SMD denotes standardised mean difference.

Seven of the eight long covid studies eligible for meta-analysis also employed measures of mood. Participants with brain fog demonstrated significantly greater scores of low mood (indicating that they were more depressed), compared with participants without brain fog (Hedge’s g=1.48, 95% CI 0.40 to 2.55, p<0.0001) (figure 4).

### Heterogeneity and risk of bias

Substantial heterogeneity was observed across studies, with I² values exceeding 80% for fatigue and mood analyses. I² was 73.9% for the analysis of cognition studies.

Visual inspection of funnel plots showed some asymmetry for cognition and fatigue outcomes, suggesting possible publication bias. Funnel plots are presented in online supplemental figures 1, 3 and 5.

Leave-one-out analyses indicated that the cognition findings were less robust, with effect sizes sensitive to several individual studies. Fatigue and mood findings were more stable, although the fatigue effect was influenced by two studies (Ortelli 2022 and Hausswirth 2023). Leave-one-out analyses are presented in online supplemental figures 2, 4 and 6) and online supplemental tables 5–7.

## Discussion

Brain fog represents a source of morbidity in a number of conditions, with little current consensus on optimal treatments. Long-term follow-up data suggest that post-COVID-19 symptoms persist in approximately 30% of patients 2 years after infection, with fatigue and cognitive impairments among the most prevalent lingering symptoms.^39^ Neurocognitive impairments have been reported in survivors of breast cancer more than 20 years after they completed chemotherapy treatment.^40^ These findings highlight the persistence of brain fog across conditions and underscore the urgent need to identify effective treatments; however, progress is hampered by the lack of consensus on how brain fog is defined and measured, making comparisons across studies and conditions difficult.

We found that researchers who have studied brain fog in long covid and in people receiving chemotherapy have used a wide variety of different neuropsychological tests; these have been used both as criteria for diagnosing brain fog, and to characterise the features of brain fog. The most common characteristics that researchers measured were cognition, fatigue and mood. In the long covid studies, which compared participants with brain fog to brain fog-free controls, we found a minor, subclinical cognitive deficit among the brain fog group compared with controls. We also found lower mood scores and increased fatigue scores in the brain fog participants. Effect sizes for mood and fatigue deficits were greater than those for cognitive deficits based on separate meta-analyses.

The range of tools we identified overlapped with those highlighted in prior reviews of cancer and long covid populations. There was substantial overlap with the tests we identified and 60 tests used to assess cognition in patients with cancer as reported in a 2023 review.^41^ FACT-Cog was not included in this review, reflecting our specific focus on studies that explicitly included a brain fog population. This review also raised concerns over the ceiling effects and the sensitivity of tests to detect mild levels of cognitive impairment, a point directly relevant to brain fog, which is often characterised by subclinical cognitive changes.

Our findings for the fatigue tests used in cancer studies were broadly consistent with a 2022 clinical practice guideline and executive summary of the assessment of cancer-related fatigue.^42^ Overlapping tests included the PROMIS, FACIT-F, the Brief Fatigue Inventory and the European Organisation for Research and Treatment of Cancer Quality of Life Questionnaire Core 30 (EORTC QLQ-C30). The guideline identified four preferred screening instruments (EORTC QLQ-C30, MD Anderson Symptom Inventory, Distress Thermometer and One-Item Fatigue Scale) and eight assessment instruments (FACIT-F, Piper Fatigue Scale—Revised, Brief Fatigue Inventory, Cancer Fatigue Scale, Fatigue Symptom Inventory, PROMIS Fatigue Short Form computerized adaptive testing (CAT), and the Multidimensional Fatigue Inventory-20). This range of available tools may contribute to the difficulty in comparing research studies. For mood, our findings were broadly consistent with a 2023 review of instruments in patients with breast cancer.^43^

Our findings of the assessment tools used in long covid were in line with previously reported studies. A study investigating how fatigue in long covid is measured identified 26 measures of fatigue, with the CFS, FSS and Fatigue Assessment Scale being the most commonly used.^44^ The authors highlighted the lack of consistency in the measures used to quantify fatigue in long covid and commented that this ‘hinders comparisons across studies and impedes progress towards improved treatment and management’, which was in line with our findings. Our findings also substantially overlapped with a review of interventions for cognitive dysfunction in long covid, which listed measures used for cognition, fatigue and mood.^45^ Furthermore, the authors also commented on the varied use of different tools, impeding comparison between studies.

An important challenge for the conduct of our meta-analysis was the wide variety of cognitive tests employed within the reviewed studies. There was more overlap in the tests used in the chemotherapy studies, where specific and validated instruments have been developed for the cancer population. These include the FACT-Cog, a self-report questionnaire to quantify subjective cognitive function in chemobrain. This consists of four subscales (perceived cognitive impairments, perceived cognitive abilities, impact on quality of life, comments from others).^32^ The FACT-Cog does not contain any items that refer specifically to cancer or cancer treatment and may therefore be suitable for assessment of subjective cognitive impairment in conditions such as long covid, fibromyalgia or chronic fatigue syndrome. Recently, Debowska *et al* have developed and validated a rating scale specifically for brain fog named the Brain Fog Scale (BFS), which is a 23-item measure with questions assessing the presence of mental fatigue, impaired cognitive acuity and confusion. The BFS was developed in response to the increasing number of patients presenting with brain fog following COVID-19 infection, although the authors recommend further studies to evaluate its use in different settings and different populations.^46^ A single assessment that is widely used across conditions and can quantify subjective impairment would have clear advantages in attempting to compare and elucidate the nature of brain fog in different populations.

We found that brain fog sufferers had statistically significant reductions in objective cognitive functioning; however, the estimated size of this was equivalent to about 1.5 points on the MoCA, which is arguably unlikely to be clinically relevant. We also found higher levels of fatigue and low mood in brain fog patients compared with healthy controls, and the effect size of these differences was larger than the effect size for the cognitive differences. In addition, leave-one-out analyses suggested that the cognition findings were less robust than the mood and fatigue findings, further supporting the idea that affective and functional symptoms may be more consistent features of brain fog than objective cognitive impairment. It is plausible that the large effect size seen for fatigue and low mood is, in fact, responsible for the relatively smaller reductions in cognition observed. Previous papers have highlighted the psychosocial mechanisms that can perpetuate symptoms in post-viral illnesses such as long covid, whereby the initial infection causes fatigue, which can lead to depressive symptoms such as avolition and hopelessness.^47^ This, in turn, can lead to reduced social participation, which can cause further depressive symptoms, which can heighten subjective cognitive difficulties, contributing to the experience of brain fog. A similar process can be envisioned for patients undergoing chemotherapy.

The terminology surrounding brain fog in the literature has been inconsistent, with multiple terms used interchangeably to describe cognitive difficulties in different contexts. In chemotherapy research, the term CRCI is often used alongside ‘chemo brain’,^48^ while in the long covid literature, terms such as post-COVID-19 cognitive disorder and postacute sequelae of SARS-CoV-2 infection appear frequently.^49 50^ These overlapping definitions contribute to variability in how brain fog has been classified and studied. Some researchers define cognitive impairment based on the presence of measurable objective cognitive deficits, whereas others rely solely on subjective complaints.^51 52^ This lack of consensus is problematic, as numerous studies have noted poor correlation between objective cognitive performance and subjective cognitive impairment.^53 54^ The inconsistency in classification is also reflected in the wide-ranging estimates of brain fog prevalence, further complicating efforts to compare findings across studies.^55 56^

A key challenge for future research is determining how the medical community should define and operationalise the term ‘brain fog’. One approach would be to adopt a broad definition that includes any patient who self-identifies as experiencing brain fog, allowing for subjective experiences to guide classification. However, as a recent review by Denno *et al* highlighted, there is a risk that the term may be hampering research by conflating separate phenomena.^17^ An alternative approach could require standardised criteria, such as a specific threshold score on an assessment like the FACT-Cog, to formally diagnose cognitive impairment.

### Limitations

Several papers that were screened did not contain a distinct brain fog or subjective cognitive impairment group and therefore could not be included in our review. However, many of these studies reported the prevalence of brain fog alongside other symptoms such as fatigue, anxiety and depression.^50 57^ While these studies could not contribute directly to our meta-analysis, they highlight a possible avenue for future research. A broader review could explore the correlation between brain fog and these related symptoms to determine whether specific patterns emerge, potentially offering further insight into the mechanisms underlying subjective cognitive impairment.

Another limitation of our meta-analysis was our restriction to long covid studies. We did not identify any chemotherapy-related studies that directly compared a group with brain fog to a group without this, limiting our ability to analyse brain fog characteristics in chemotherapy and compare these with COVID-19 brain fog. Several chemotherapy studies instead focused on intervention trials, comparing a brain fog group receiving treatment to a brain fog control group.^58^,^60^

The inability to compare the characteristics of brain fog between patients with long covid and those receiving chemotherapy was one of our original aims but could not be achieved. While this represents a key limitation of the study, it reflects a major gap in the literature: namely, the lack of research within chemotherapy populations that directly compares those who develop brain fog with those who do not. Addressing this gap should be a priority for future studies, as it would enable comparison of brain fog profiles between conditions.

We were able to perform meta-analyses of the long covid studies, as several compared groups with and without brain fog. However, the majority of these studies compared long covid patients with brain fog versus healthy controls rather than long covid patients without brain fog. As such, some of the observed differences may reflect the broader effects of COVID-19 rather than deficits specifically attributable to brain fog. Notably, one of our main findings was that the overall cognitive deficit was small; it is likely that including more studies with disease-matched controls (long covid patients without brain fog) would show an even smaller effect. Future studies directly comparing brain fog and non-brain fog long covid patients are needed.

Finally, there were high levels of heterogeneity in the studies included in our meta-analyses of long covid, reflecting the variability across study populations and methodologies. As touched on already, this may in part reflect the lack of a universal definition of brain fog: in some studies individuals were categorised as having brain fog if they self-reported this as a symptom, whereas in other studies, objective cognitive tests were used to identify cases. The variety of assessment tools employed may also have contributed, along with variation in the severity and timing of the original COVID-19 infection, increasing heterogeneity in cognition, mood and fatigue. As a result, pooled effect sizes should be interpreted with caution, and greater standardisation in future research is needed. Additionally, sensitivity analyses indicated that pooled cognition and fatigue effects were partly driven by a small number of influential studies, further emphasising the need for careful interpretation.

### Conclusion

Research into the causes, course and management of brain fog faces several challenges, including heterogeneity of the condition, inconsistent terminology used and a lack of a universally agreed definition. While some authors have considered physical causes for brain fog, including neuroinflammation, neuronal cell death, blood–brain barrier dysfunction and mitochondrial dysfunction, we observed a greater association with fatigue and low mood compared with objective cognitive impairment. This is consistent with a common finding in the literature that objective and subjective cognitive impairment measures are not strongly correlated,^54^ and more studies in long covid that measure subjective cognitive impairment are needed. Researchers should be open-minded about potential psychosocial aetiologies of brain fog. Studies that compare groups with brain fog with those from the same population (eg, COVID-19, chemotherapy) who do not experience brain fog are needed. Furthermore, the field is still in need of a universally agreed definition of brain fog and criteria for diagnosing brain fog. Further research should focus on proposing such a definition and criteria.

## Supplementary material

10.1136/bmjment-2025-301969 online supplemental file 1

## Data Availability

Data are available on reasonable request.
