# Supplementary material for: Brain fog with long covid and chemotherapy: systematic review and meta-analysis
Source: BMJ Ment Health. 2025 Dec 17;28(1):e301969. doi: 10.1136/bmjment-2025-301969 (PMC12716526; doi:10.1136/bmjment-2025-301969)
Supplement: online supplemental file 1 [file bmjment-28-1-s001.pdf]

Supplementary Table 1:
Tally of neuropsychological tests used in Long COVID studies

**Cognition**		**Function**		**Mood**	
Montreal Cognitive Assessment	17	Fatigue Severity Scale	11	Beck Depression Inventory-II	9
Trail Making Test	9	Chalder Fatigue Scale	6	Hospital Anxiety and Depression Scale	7
Stroop Color and Word Test (SCWT)	8	World Health Organization Quality of Life - BREF	3	Patient Health Questionnaire-9	6
Digit Span	6	EuroQol 5-Dimension	3	General Anxiety Disorder-7	5
Symbol Digit Modalities Test	4	Pittsburgh Sleep Quality Index	3	Impact of Event Scale – Revised	4
Verbal Fluency	4	6-minute walk test (6MWT)	3	Beck Anxiety Inventory	3
Boston Naming Test	4	Short Form 36 Health Survey	2	State-Trait Anxiety Inventory	2
Rey Auditory Verbal Learning Test	3	International Physical Activity Questionnaire - Short Form	2	Posttraumatic Stress Disorder Checklist for DSM-5	2
Wechsler Adult Intelligence Scale – Fourth Edition	3	Modified Medical Research Council (mMRC) scale score for dyspnea	2	Patient-Reported Outcomes Measurement Information System - Depression	2
Rey-Osterrieth Complex Figure Test	3	post-COVID functional scale	2	Patient-Reported Outcomes Measurement Information System - Anxiety	2
Animal fluency	3	Multidimensional Fatigue	2	Profile of Mood States	2

		Inventory			
Mini Mental State Exam	3	Functional Assessment of Chronic Illness Therapy - Fatigue	1	Depression Anxiety Stress Scales	1
Patient-Reported Outcomes Measurement Information System (PROMIS) - Cognition	2	PROMIS Quality of life Fatigue	1	Center for Epidemiologic Studies Depression Scale	1
Paced Auditory Serial Addition Test	2	Canadian Occupational Performance Measure	1	Positive and Negative Affect Schedule	1
Paced Auditory Serial Addition Test	2	Fatigue Rating Scale	1	Insomnia Severity Index	1
Judgment of Line Orientation	2	Pulmonary function tests	1	Hamilton Anxiety Rating Scale	1
Controlled Oral Word Association Test	2	handgrip endurance at 50%max	1	Smyptoms Checklist 90	1
Corsi Block-Tapping	2	HGS maximum handgrip strength	1	Zung Self-Rating Depression Scale	1
Choice response time	2	Epworth Sleepiness Scale	1	Zung Self-Rating Anxiety Scale	1
Digit Symbol Substitution Test,	2	Visual Analog Scale	1	Post-COVID-19 Symptoms Assessment Questionnaire	1
Word Accentuation Test	1	Spiegel Sleep Quality questionnaire	1	General Anxiety Disorder-2	1
Hopkins Verbal Learning Test	1	Brunnsviken Brief Quality of Life scale	1	Wellbeing scale	1

Wechsler Adult Intelligence Scale – Third Edition	1	Post-COVID-19 Symptoms Assessment Questionnaire (A-PASC) Functional	1		
Grooved pegboard	1	assessment of Quality of Life - six domains	1		
Wechsler Memory Scale	1	Modified Fatigue Impact Scale	1		
d2 Test of Attention	1	Insomnia Severity Scale	1		
Perceived Cognitive Difficulties Evaluation	1	Short Form 12 Health Survey	1		
Subjective cognitive complaints questionnaire	1	Subjective Whole Body Fatigue	1		
McNair self-questionnaire	1	Brief Fatigue Inventory	1		
European AIDS Clinical Society cognitive screening questions	1	Fatigue Scale for Motor and Cognitive Functions	1		
Mini mental state exam 3	1	Perceived Stress Scale	1		
Brief Visuospatial Memory Test – Revised	1				
Wide Range Achievement Test – Fourth Edition.	1				
Delis–Kaplan Executive Function System	1				

Conners Continuous Performance Test – Third Edition	1				
Frontal Assessment Battery	1				
Word List	1				
Modified five point test	1				
Perceived Cognitive Difficulties Scale	1				
Mehrfachwahl-Wortschatz-Intelligenztest, Version B,	1				
CNS Vital Signs – Neurocognitive Index	1				
Conners' Continuous Performance Test, Second Edition.	1				
Neuropsychological Assessment Battery (NAB)	1				
Pursuit Rotor	1				
Simon Task	1				
Pattern Comparison	1				
Block Design Test	1				
Coding	1				
Symbol Search	1				
Post-COVID-19 Symptoms Assessment Questionnaire (A-PASC) Cognitive	1				

Fatigue Impact Scale (Cognitive)	1				
FLEI Mental Ability Questionnaire	1				
Free and Cued Selective Reminding Test	1				
Brain fog severity scale	1				
5 Objects Test	1				
Benson Complex Figure Test	1				
Digit Symbol Substitution Test	1				
CERAD Word List Memory Test	1				
Craft Story Test	1				
Cognitron	1				
Cognitive Chalder Score	1				
Repeatable Battery for the Assessment of Neuropsychological Status	1				
Oral Trails	1				

Supplementary Table 2:
Tally of neuropsychological tests used in chemotherapy studies

Cognition		Function		Mood	
Functional Assessment of Cancer Therapy - Cognition	15	Patient-Reported Outcomes Measurement Information System	3	Beck Depression Inventory-II	4
Trail Making Test	7	Functional Assessment of Cancer Therapy - Fatigue	3	State-Trait Anxiety Inventory	3
Digit span	7	Functional Assessment of Chronic Illness Therapy	2	Patient Health Questionnaire-9	3
Rey Auditory Verbal Learning Test	5	Functional Assessment of Cancer Therapy - Breast	2	Center for Epidemiologic Studies Depression Scale	2
Mini Mental State Exam	3	Quality of Life Questionnaire - Core 30	1	Depression Anxiety Stress Scales	1
Hopkins Verbal Learning Test	3	Short Form 36 Health Survey	1	Beck Anxiety Inventory	1
Controlled Oral Word Association Test	3	Brief Fatigue Inventory	1	Clinical Assessment of Depression (CAD)	1
Stroop Color and Word Test (SCWT)	3	Functional Assessment of Cancer Therapy - Anemia	1	Hospital Anxiety and Depression Scale	1
Verbal Fluency	2	Functional Assessment of Cancer Therapy - General	1	General Health Questionnaire-12	1
Rey-Osterrieth Complex Figure Test	2	MD Anderson Symptom Inventory - Multiple	1	Perceived Stress Scale-14	1

		Myeloma			
Montreal Cognitive Assessment	2	Checklist Individual Strength - Fatigue subscale	1	General Anxiety Disorder-7	1
CogState	2	Pittsburgh Sleep Quality Index	1	Distress Thermometer (DT)	1
Cognitive Failures Questionnaire	2	Canadian Occupational Performance Measure	1	Profile of Mood States	1
Wechsler Adult Intelligence Scale – Fourth Edition	1	Resilience Assessment Profile	1	Brief Symptom Inventory 18	1
Symbol Digit Modalities Test	1			Whiteley-7 Scale	1
Patient-Reported Outcomes Measurement Information System	1			Symptom Checklist 90 - Anxiety	1
Paced Auditory Serial Addition Test	1				
Letter Fluency	1				
High Sensitivity Cognitive Screen	1				
Headminder	1				
Grooved pegboard	1				
Dysexecutive Questionnaire	1				
Delis–Kaplan Executive Function System	1				
d2 Test of Attention	1				
Cognitive Estimation Task	1				
Coghealth	1				

Choice reaction time test	1				
Clock Drawing Test	1				
Animal Fluency	1				
Auditory Consonant Trigrams Test	1				
Continuous Performance Test Task #2	1				
MemTrax	1				

Supplementary Table 3:
Chemotherapy studies' demographic details
BF+ve: subjects had brain fog (or equivalent term) but did not receive intervention in study
BF-ve: subjects had received either chemotherapy or had recovered from COVID, but did not have brain fog (or equivalent term)
HC: Health controls

Author	Single group / comparison	Total no.	No. brain fog	Comparison group	No. Compariso n group	Average age BF	Average age comparison groups	% Female brain fog	% Female comparison group	Quality Assessm ent
Alvarez 2013 [1]	Single group	23	23			56		100		Medium
Bray 2016 [2]	Comparison	242	121	BF+ve	121	52	54	96	94	High
Campbell 2017 [3]	Comparison	19	10	BF+ve	9	53	51	100	100	Medium
Damholdt 2016 [4]	Comparison	157	94	BF+ve	63	55	55	100	100	High
Ding 2020 [5]	Comparison	74	34	BF+ve	40	51	51	100	100	Medium
Dos Santos 2020 [6]	Comparison	167	Group A 55	Group B(BF+ve) Group C (BF+ve)	Group B: 56 Group C: 56	52	Group B: 50.9 Group C: 50.7	96	Group B: 94.6 Group C: 96.4	High
Kesler et al. 2023 [7]	Comparison	160	80	HC	80	Biotype 1: 49 Biotype 2: 52 Biotype 3: 52	49	100	100	Medium

Koevoets 2022 [8]	Comparison	181	91	BF+ve	90	52	53	100	100	High
Lawrence 2015 [9]	Comparison	62	31	BF+ve	31	56	56	100	100	High
Liang 2018 [10]	Single group	12	12			46		100		Medium
Ma 2024 [11]	Comparison	41	18	BF-ve	23	50	45	100	100	High
Milbury 2013 [12]	Comparison	47	23	BF+ve	24	53	54	100	100	Medium
Piccirillo et al., 2015 [13]	Comparison	28	15	BF-ve	13	53	52	100	100	Medium
Rodriguez-Wolfe 2019 [14]	Single group	6	6					100		Medium
Sideroff 2022 [15]	Single group	10	9			57		100		Medium
Tchen 2003 [16]	Comparison	291	100	HC	100	48	47	100	100	Medium
Tong 2018 [17]	Comparison	80	39	BF+ve	36	43	42	100	100	Medium
Van de Gucht 2020 [18]	Comparison	33	18	BF+ve	15	44	47	100	100	Medium
Vardy 2006 [19]	Single group	31	31			52		94		Medium
Vega 2022 [20]	Comparison	25	12	BF+ve	13	55	53	100	100	Medium

Supplementary Table 4:
COVID studies' demographic details

Author's Surname and Year	Single group / comparison	Total no.	No. brain fog	Comparison group	No. Comparison group	Average age BF	Average age comparison groups	% Female brain fog	% Female comparison group	Quality score
Arbula 2024 [26]	Comparison	60	33	HC	27	54	57	76	63	High
Ariza 2024 [27]	Comparison	492	ICU-PCC: 81<br>Hospital-PCC: 80<br>Mild-PCC: 207	HC	124	ICU-PCC: 53<br>Hospital-PCC: 53<br>Mild-PCC: 48	47	ICU-PCC: 47<br>Hospital-PCC: 50<br>Mild-PCC: 80	75	High
Ariza 2023 [28]	Comparison	428	319	HC	109	ICU patients: 51.9, Hospitalised patients: 52.69, Mild patients: 46.21	46	ICU patients: 44.2, Hospitalised patients: 50, Mild patients: 77	77	Medium
Babiloni 2024 [29]	Comparison	51	27	HC<br>BF-ve	HC 15<br>BF-ve 9	59.3	HC 59.1<br>BF-ve 61.4	44	HC 47<br>BF-ve 33	Medium
Bland 2024 [30]	Comparison	162	50	BF-ve<br>HC	BF-ve 59<br>HC 53	41.66	BF-ve 38.12<br>HC 41.98	58	BF-ve 62%<br>HC 47%	High
Bonfim 2024 [31]	Comparison	630	149	BF-ve	481	33	30	80.5	62.2	Medium

Bulla 2023 [32]	Comparison	66	32	BF-ve HC	BF-ve 16 HC 18	54	BF-ve 47 HC 44	75	BF-ve 75 HC 72	Medium
Calabria 2022 [33]	Single group	136	136			52		64		Medium
Cristillo 2022 [34]	Comparison	132	25	BF-ve	107	69	65	29	30	High
Delgado-Alonso 2023 [35]	Single group	170	170			49		73		Medium
Deodato 2024[36]	Comparison	20	10	BF+ve	10	50	56	70	90	Medium
Furlanis 2023[37]	Single group	20	20			49		70		Medium
Guttuso 2024 [38]	Comparison	50	24	BF+ve	26	62.3	55	42	46	High
Hammerle 2023 [39]	Comparison	127	68	BF-ve	59	42 (Whole cohort)		76 (Whole cohort)		Medium
Hanson 2023 [40]	Comparison	92	48	BF-ve HC	BF-ve 20 HC 24	45	BF-ve 38.7 HC 30	71	BF-ve 70 HC 63	Medium
Hausswirth 2023 [41]	Comparison	49	17	BF+ve HC	BF+ve 17 HC 15	47	BF+ve 49 HC 46	76	BF+ve 71 HC 67	Low
Hu 2024 [42]	Comparison	19	2	BF+ve	17	44	54.9			Medium

Jennings 2022 [43]	Comparison	108	71	BF-ve	37	46	46	77	60	Medium
Klírová 2024 [44]	Comparison	33	16	BF-ve	17	44	40	69	71	High
Krishnan 2022 [45]	Single group	20	20			45		90		Medium
Lam 2023 [46]	Comparison	1,642	985	BF-ve	657					High
LeGoff 2023 [47]	Single group	64	64			48		55		Low
Lynch 2022 [48]	Single group	60	60			41		"about two thirds"		Medium
Manganotti 2023 [49]	Comparison	34	18	HC	16	55	Not stated	67	Not stated	Medium
McGregor 2024 [50]	Comparison	585	298	BF+ve	287	56	56	52	50	High
Morin 2021 [51]	Single group	478	478			60.9 years		42		Medium
Nielsen 2022 [52]	Single group	448	448			47		73		Medium
Niemczak 2025 [53]	Comparison	20	10	HC	10	47.9	49.5	80	80	Medium

Orfei 2021 [54]	Comparison	441	177	HC<br>PBF	HC 147<br>PBF 117	46	HC 48<br>PBF 48	50	HC 27<br>PBF 37	Medium
Ortelli 2023 [55]	Comparison	36	18	HC	18	41	48	56	44	Medium
Ortelli 2022[1] [56]	Comparison	89	67	HC	22	50	46	75	50	Medium
Rabaiotti 2023 [57]	Comparison	64	Mild: 36<br>Moderate: 18<br>Severe: 2	HC	8	Mild: 66<br>Moderate: 72<br>Severe: 77	62	Mild: 67<br>Moderate: 33<br>Severe: 50	100	Medium
Rossato 2021 [58]	Single group	201	201			48		61		Medium
Ruzicka 2024 [59]	Comparison	315	56	None, Mild, or Moderate Cognitive symptoms	259	40.5	41	66.1	61	High
Sag 2023 [60]	Comparison	31	16	HC	15	37	37	75	Not stated	Medium
Sathyamoorthy 2024 [61]	Comparison	80	38	NCI	42	49	50	58	64	Medium
Schild 2023 [62]	Single group	58	52			46		60		Low

[1] In the paper *Altered motor cortex physiology and dysexecutive syndrome in patients with fatigue and cognitive difficulties after mild COVID-19* by Ortelli et al in table 1 Beck Depression Inventory-II (BDI-II) scores are reported as: Patients: 1.86 (2.70), Controls: 16.34 (8.26), however the authors state in the text: "BDI-II showed significantly higher scores in patients than in [healthy controls]." In correspondence with ourselves, Ortelli confirmed there had been an error in the table and the correct BDI-II scores were Patients: 16.34 (8.26), Controls: 1.86 (2.70) - these were the figures we included in the meta-analysis.

Victoria 2024 [63]	Comparison	98	49	BF+ve	49	49.5	46.8	78	80	Medium
Whiteside 2022 [64]	Single group	49	49			50		84		Medium
Wright 2024 [65]	Single group	13	13			47.3		85		Medium

Supplementary Figure 1:
Funnel plot for cognitive outcomes in Long COVID meta-analysis

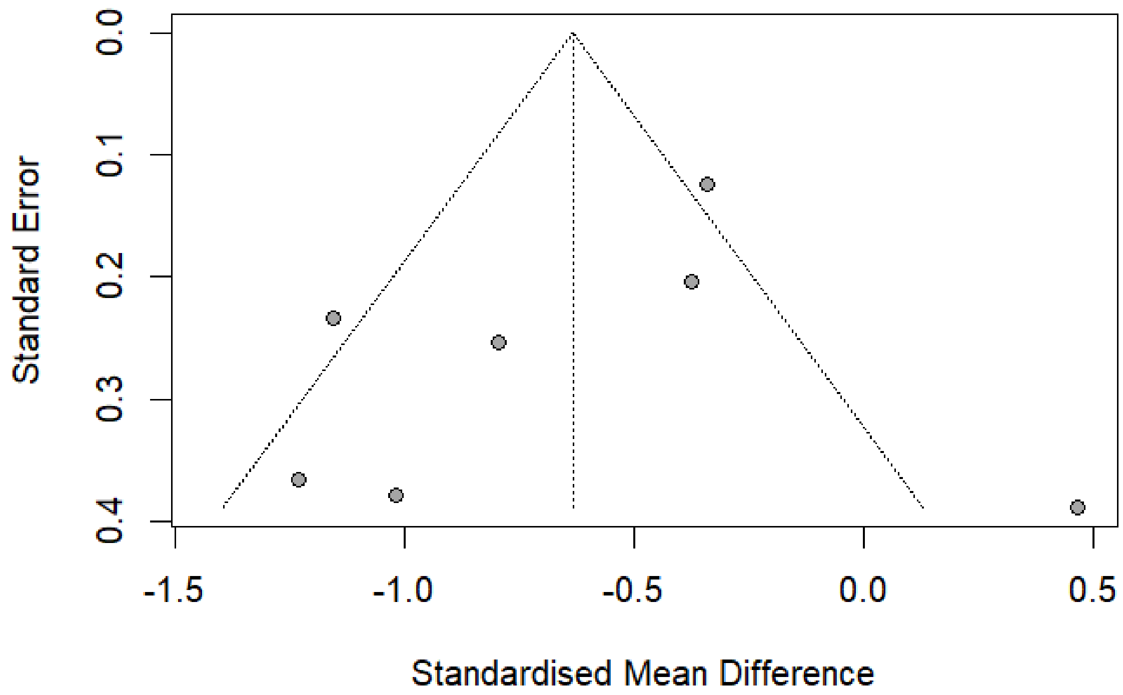

Supplementary Figure 2:
Leave-one-out plots for cognitive outcomes in Long COVID meta-analysis

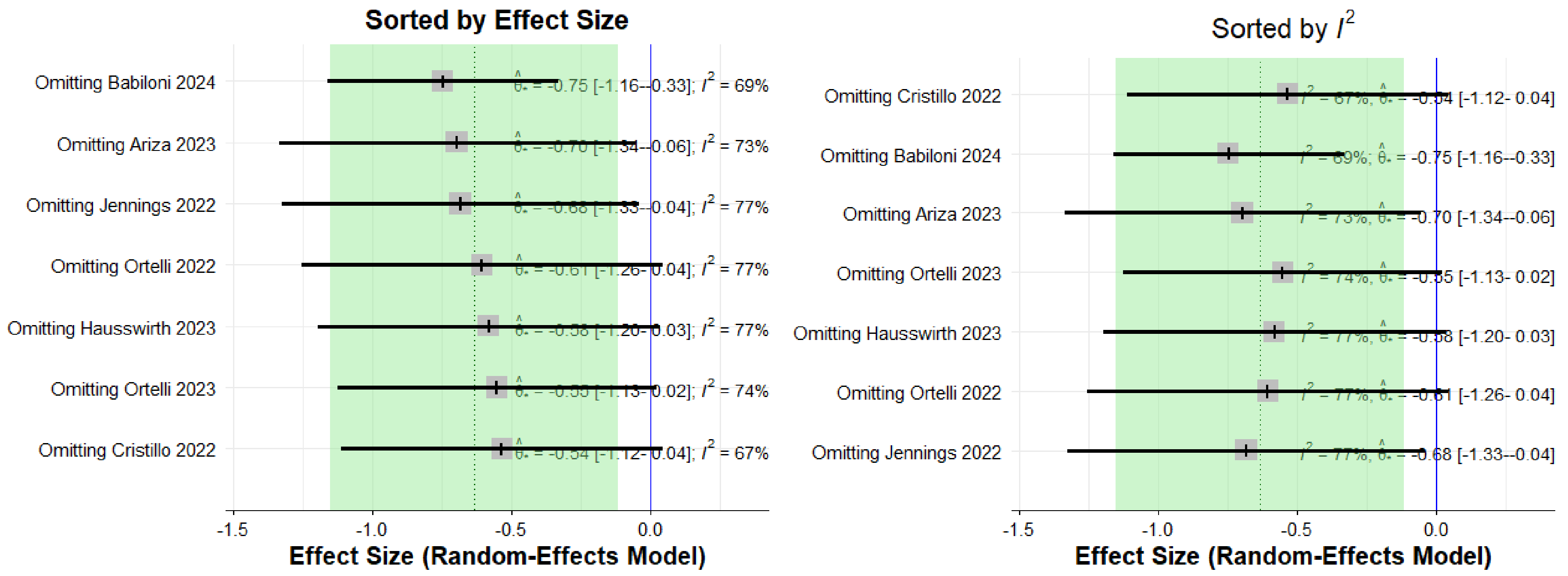

Supplementary Table 5:
Leave-one-out influence analysis for cognition outcomes in Long COVID meta-analysis

Study Omitted	Effect Size (Hedge's g)	95% CI (Lower)	95% CI (Upper)	I²
Cristillo 2022	-0.536	-1.115	0.043	0.668
Babiloni 2024	-0.749	-1.164	-0.333	0.687
Ariza 2023	-0.698	-1.338	-0.057	0.726

Ortelli 2023	-0.553	-1.127	0.02	0.74
Hausswirth 2023	-0.583	-1.198	0.032	0.765
Ortelli 2022	-0.607	-1.256	0.042	0.771
Jennings 2022	-0.684	-1.326	-0.042	0.774

Supplementary Figure 3:
Funnel plot for fatigue outcomes in Long COVID meta-analysis

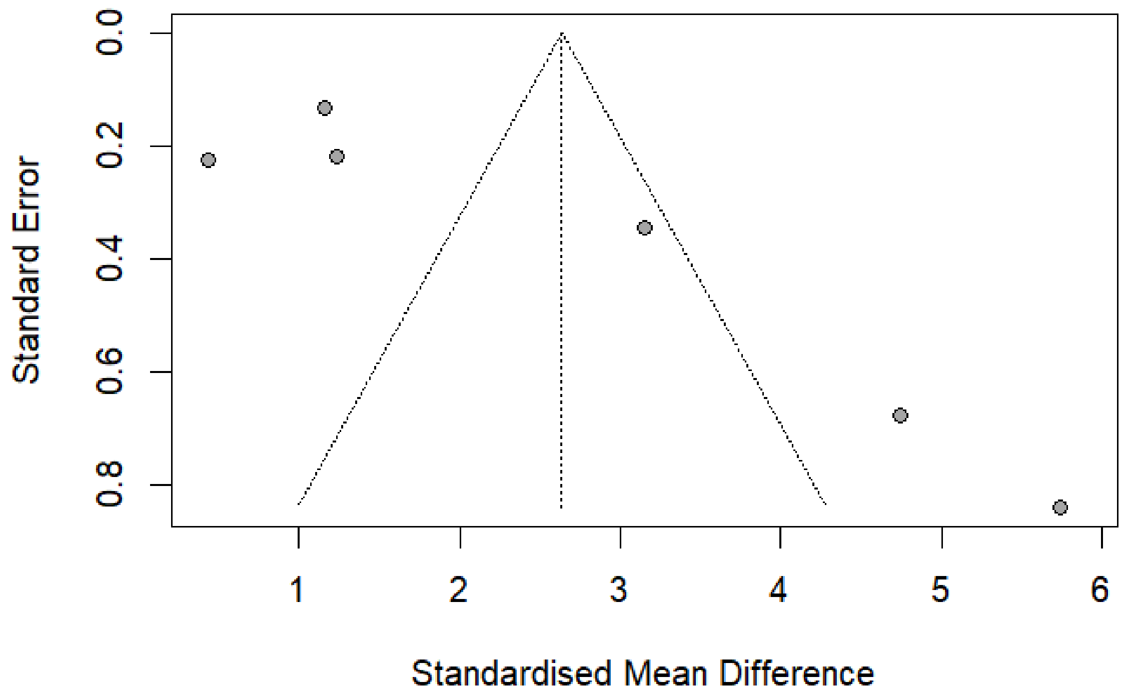

Supplementary Figure 4:
Leave-one-out plots for fatigue outcomes in Long COVID meta-analysis

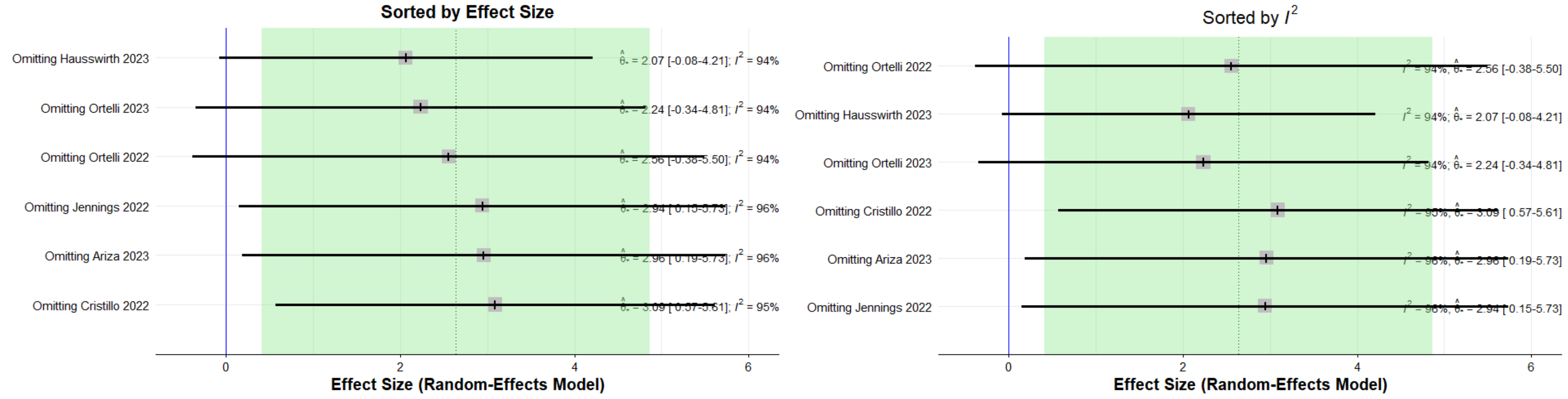

Supplementary Table 6:
Leave-one-out influence analysis for fatigue outcomes in Long COVID meta-analysis

Study Omitted	Effect Size (Hedge's g)	95% CI (Lower)	95% CI (Upper)	I²
Ortelli 2022	2.555	-0.385	5.496	0.941
Hausswirth 2023	2.065	-0.077	4.207	0.943
Ortelli 2023	2.235	-0.344	4.815	0.945
Cristillo 2022	3.089	0.568	5.61	0.95
Ariza 2023	2.96	0.188	5.733	0.958
Jennings 2022	2.944	0.153	5.735	0.959

Supplementary Figure 5:
Funnel plot for mood outcomes in Long COVID meta-analysis

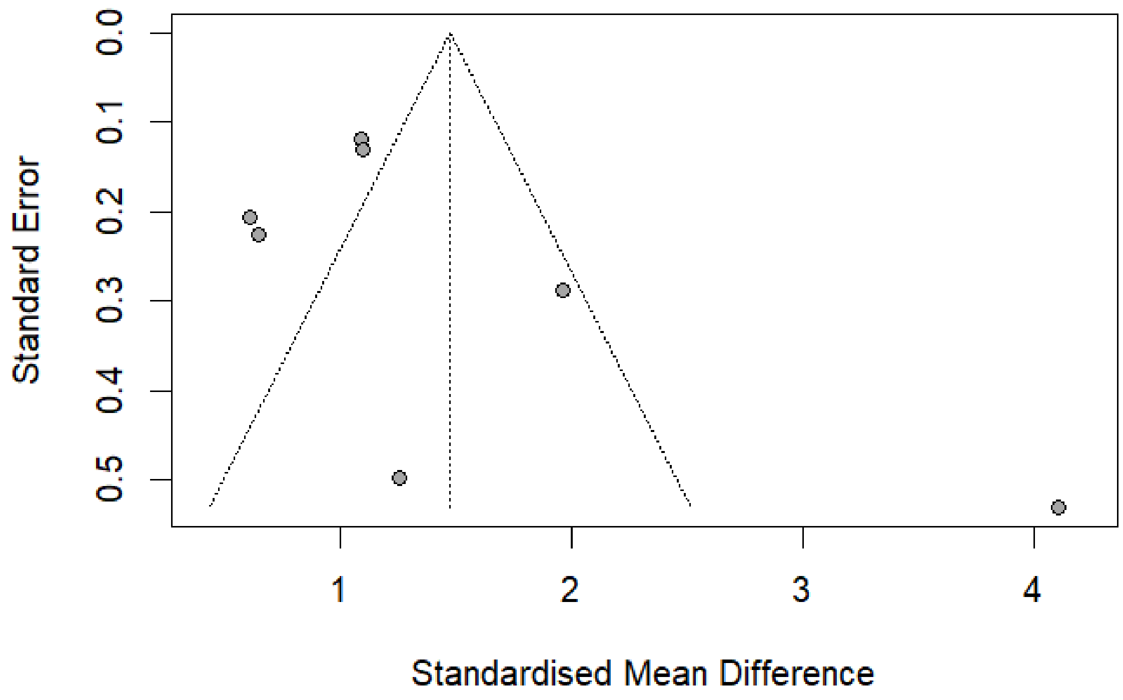

Supplementary Figure 6:
Leave-one-out plots for mood outcomes in Long COVID meta-analysis

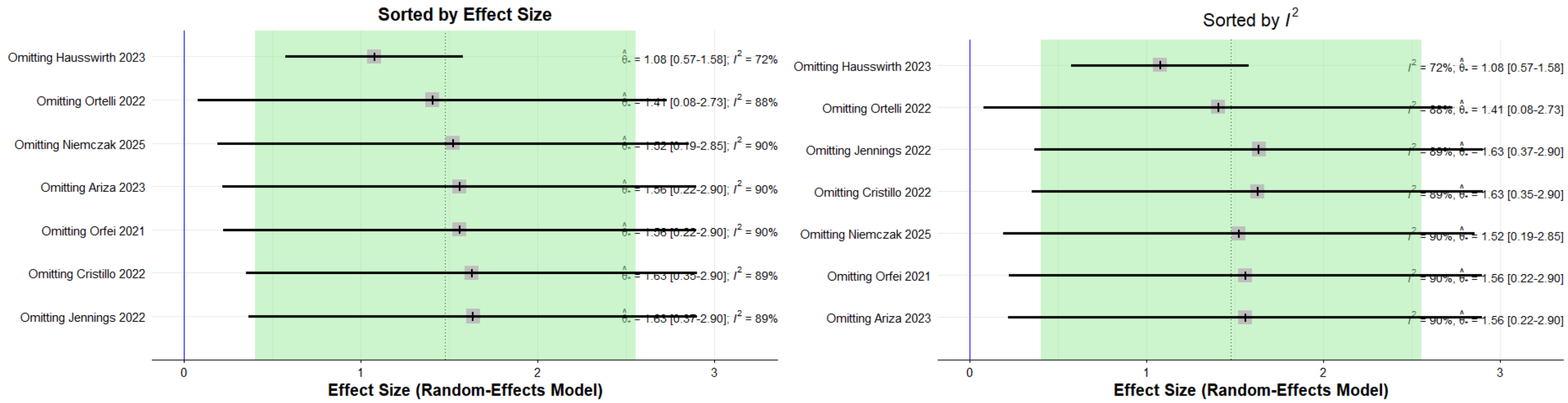

Supplementary Table 7:
Leave-one-out influence analysis for mood outcomes in Long COVID meta-analysis

Study Omitted	Effect Size (Hedge's g)	95% CI (Lower)	95% CI (Upper)	I²
Hausswirth 2023	1.076	0.575	1.578	0.724
Ortelli 2022	1.406	0.08	2.731	0.879
Jennings 2022	1.634	0.367	2.902	0.887
Cristillo 2022	1.627	0.352	2.903	0.892
Orfei 2021	1.559	0.22	2.897	0.901
Ariza 2023	1.557	0.218	2.896	0.901
Niemczak 2025	1.522	0.188	2.855	0.901

1. Alvarez J, Meyer FL, Granoff DL, et al. The effect of EEG biofeedback on reducing postcancer cognitive impairment. *Integr Cancer Ther* 2013; 12: 475–487.

2. Bray VJ, Dhillon HM, Bell ML, et al. Evaluation of a Web-Based Cognitive Rehabilitation Program in Cancer Survivors Reporting Cognitive Symptoms After Chemotherapy. *J Clin Oncol* 2017; 35: 217–225.

3. Campbell KL, Kam JWY, Neil-Sztramko SE, et al. Effect of aerobic exercise on cancer-associated cognitive impairment: A proof-of-concept RCT. *Psychooncology* 2018; 27: 53–60.

4. Damholdt MF, Mehlsen M, O'Toole MS, et al. Web-based cognitive training for breast cancer survivors with cognitive complaints-a randomized controlled trial. *Psychooncology* 2016; 25: 1293–1300.

5. Ding K, Zhang X, Zhao J, et al. Managing Cancer and Living Meaningfully (CALM) Intervention on Chemotherapy-Related Cognitive Impairment in Breast Cancer Survivors. *Integr Cancer Ther* 2020; 19: 1534735420938450.

6. Dos Santos M, Hardy-Léger I, Rigal O, et al. Cognitive rehabilitation program to improve cognition of cancer patients treated with chemotherapy: A 3-arm randomized trial. *Cancer* 2020; 126: 5328–5336.

7. Mulholland MM, Prinsloo S, Kvale E, et al. Behavioral and biologic characteristics of cancer-related cognitive impairment biotypes. *Brain Imaging Behav* 2023; 17: 320–328.

8. Koevoets EW, Schagen SB, de Ruiter MB, et al. Effect of physical exercise on cognitive function after chemotherapy in patients with breast cancer: a randomized controlled trial (PAM study). *Breast Cancer Res* 2022; 24: 36.

9. Lawrence JA, Griffin L, Balcueva EP, et al. A study of donepezil in female breast cancer survivors with self-reported cognitive dysfunction 1 to 5 years following adjuvant chemotherapy. *J Cancer Surviv* 2016; 10: 176–184.

10. Liang MI, Erich B, Bailey C, et al. Emerging From the Haze: A Pilot Study Evaluating Feasibility of a Psychoeducational Intervention to Improve Cancer-Related Cognitive Impairment in Gynecologic Cancer Survivors. *J Palliat Care* 2019; 34: 32–37.

11. Ma Y, Chai W, Bu D, et al. Toward better understanding and management of chemobrain: the potential utilities of the MemTrax memory test. *BMC Womens Health* 2024; 24: 406.

12. Milbury K, Chaoul A, Biegler K, et al. Tibetan sound meditation for cognitive dysfunction: results of a randomized controlled pilot trial. *Psychooncology* 2013; 22: 2354–2363.

13. Piccirillo JF, Hardin FM, Nicklaus J, et al. Cognitive impairment after chemotherapy related to atypical network architecture for executive control. *Oncology* 2015; 88: 360–368.

14. Rodriguez-Wolfe M, Anglade D, Gattamorta KA, et al. Individualized Piano Instruction for Improving Cognition in Breast Cancer Survivors. *Oncol Nurs Forum* 2019; 46: 605–615.

15. Sideroff S, Wellisch D, Yarema V. A neurotherapy protocol to remediate cognitive deficits after adjuvant chemotherapy: a pilot study. *J Complement Integr Med* 2023; 20: 447–456.

16. Tchen N, Juffs HG, Downie FP, et al. Cognitive function, fatigue, and menopausal symptoms in women receiving adjuvant chemotherapy for breast cancer. *J Clin Oncol* 2003; 21: 4175–4183.

17. Tong T, Pei C, Chen J, et al. Efficacy of Acupuncture Therapy for Chemotherapy-Related Cognitive Impairment in Breast Cancer Patients. *Med Sci Monit* 2018; 24: 2919–2927.

18. Van der Gucht K, Ahmadoun S, Melis M, et al. Effects of a mindfulness-based intervention on cancer-related cognitive impairment: Results of a randomized controlled functional magnetic resonance imaging pilot study. *Cancer* 2020; 126: 4246–4255.

19. Vardy J, Wong K, Yi Q-L, et al. Assessing cognitive function in cancer patients. *Support Care Cancer* 2006; 14: 1111–1118.

20. Vega JN, Albert KM, Mayer IA, et al. Subjective cognition and mood in persistent chemotherapy-related cognitive impairment. *J Cancer Surviv* 2022; 16: 614–623.

21. Vitor T, Kozasa EH, Bressan RA, et al. Impaired brain dopamine transporter in chemobrain patients submitted to brain SPECT imaging using the technetium-99m labeled tracer TRODAT-1. *Ann Nucl Med* 2019; 33: 269–279.

22. Wang J, Lian C-L, Zheng H, et al. Cognitive dysfunction in patients with nasopharyngeal carcinoma after induction chemotherapy. *Oral Oncol* 2020; 111: 104921.

23. Wolf TJ, Doherty M, Kallogjeri D, et al. The Feasibility of Using Metacognitive Strategy Training to Improve Cognitive Performance and Neural Connectivity in Women with Chemotherapy-Induced Cognitive Impairment. *Oncology* 2016; 91: 143–152.

24. Yao S, Ding K, Liu S, et al. The Managing Cancer and Living Meaningfully (CALM) Intervention Alleviates Chemotherapy-Related Cognitive Impairment in Patients with Breast Cancer by Modulating Pan-Immune-Inflammation Values. *Integr Cancer Ther* 2022; 21: 15347354221140498.

25. Yao S, Zhu Q, Zhang Q, et al. Managing Cancer and Living Meaningfully (CALM) alleviates chemotherapy related cognitive impairment

(CRCI) in breast cancer survivors: A pilot study based on resting-state fMRI. *Cancer Med* 2023; 12: 16231–16242.

26. Arbula S, Pisanu E, Bellavita G, et al. Insights into attention and memory difficulties in post-COVID syndrome using standardized neuropsychological tests and experimental cognitive tasks. *Sci Rep* 2024; 14: 4405.

27. Ariza M, Cano N, Segura B, et al. Cognitive and emotional predictors of quality of life and functioning after COVID-19. *Ann Clin Transl Neurol* 2024; 11: 302–320.

28. Ariza M, Cano N, Segura B, et al. COVID-19 severity is related to poor executive function in people with post-COVID conditions. *J Neurol* 2023; 270: 2392–2408.

29. Babiloni C, Gentilini Cacciola E, Tucci F, et al. Resting-state EEG rhythms are abnormal in post COVID-19 patients with brain fog without cognitive and affective disorders. *Clin Neurophysiol* 2024; 161: 159–172.

30. Bland AR, Barraclough M, Trender WR, et al. Profiles of objective and subjective cognitive function in Post-COVID Syndrome, COVID-19 recovered, and COVID-19 naïve individuals. *Sci Rep* 2024; 14: 13368.

31. Bonfim LPF, Correa TR, Freire BCC, et al. Post-COVID-19 cognitive symptoms in patients assisted by a teleassistance service: a retrospective cohort study. *Front Public Health* 2024; 12: 1282067.

32. Bulla R, Rossi L, Furlanis G, et al. A likely association between low mannan-binding lectin level and brain fog onset in long COVID patients. *Front Immunol* 2023; 14: 1191083.

33. Calabria M, García-Sánchez C, Grunden N, et al. Post-COVID-19 fatigue: the contribution of cognitive and neuropsychiatric symptoms. *J Neurol* 2022; 269: 3990–3999.

34. Cristillo V, Pilotto A, Piccinelli SC, et al. Predictors of ‘brain fog’ 1 year after COVID-19 disease. *Neurol Sci* 2022; 43: 5795–5797.

35. Delgado-Alonso C, Díez-Cirarda M, Pagán J, et al. Unraveling brain fog in post-COVID syndrome: Relationship between subjective cognitive complaints and cognitive function, fatigue, and neuropsychiatric symptoms. *Eur J Neurol* 2025; 32: e16084.

36. Deodato M, Qualizza C, Martini M, et al. Efficacy of dual-task augmented reality rehabilitation in non-hospitalized adults with self-reported long COVID fatigue and cognitive impairment: a pilot study. *Neurol Sci* 2024; 45: 1325–1333.

37. Furlanis G, Buoite Stella A, Biaduzzini F, et al. Cognitive deficit in post-acute COVID-19: an opportunity for EEG evaluation? *Neurol Sci* 2023; 44: 1491–1498.

38. Guttuso T Jr, Zhu J, Wilding GE. Lithium aspartate for long COVID fatigue and cognitive dysfunction: A randomized clinical trial: A randomized clinical trial. *JAMA Netw Open* 2024; 7: e2436874.

39. Hammerle MB, Sales DS, Pinheiro PG, et al. Cognitive Complaints Assessment and Neuropsychiatric Disorders After Mild COVID-19 Infection. *Arch Clin Neuropsychol* 2023; 38: 196–204.

40. Hanson BA, Visvabharathy L, Orban ZS, et al. Plasma proteomics show altered inflammatory and mitochondrial proteins in patients with neurologic symptoms of post-acute sequelae of SARS-CoV-2 infection. *Brain Behav Immun* 2023; 114: 462–474.

41. Hausswirth C, Schmit C, Rougier Y, et al. Positive Impacts of a Four-Week Neuro-Meditation Program on Cognitive Function in Post-Acute Sequelae of COVID-19 Patients: A Randomized Controlled Trial. *Int J Environ Res Public Health*; 20. Epub ahead of print 11 January 2023. DOI: 10.3390/ijerph20021361.

42. Hu WT, Kaluzova M, Dawson A, et al. Clinical and CSF single-cell profiling of post-COVID-19 cognitive impairment. *Cell Rep Med* 2024; 5: 101561.

43. Jennings G, Monaghan A, Xue F, et al. Comprehensive Clinical Characterisation of Brain Fog in Adults Reporting Long COVID Symptoms. *J Clin Med*; 11. Epub ahead of print 15 June 2022. DOI: 10.3390/jcm11123440.

44. Klírová M, Adamová A, Biačková N, et al. Transcranial direct current stimulation (tDCS) in the treatment of neuropsychiatric symptoms of long COVID. *Sci Rep* 2024; 14: 2193.

45. Krishnan K, Miller AK, Reiter K, et al. Neurocognitive Profiles in Patients With Persisting Cognitive Symptoms Associated With COVID-19. *Arch Clin Neuropsychol* 2022; 37: 729–737.

46. Lam GY, Damant RW, Ferrara G, et al. Characterizing long-COVID brain fog: a retrospective cohort study. *J Neurol* 2023; 270: 4640–4646.

47. LeGoff DB, Lazarovic J, Kofeldt M, et al. Neurocognitive and Symptom Validity Testing for Post-COVID-19 Condition in a Workers Compensation Context. *J Occup Environ Med* 2023; 65: 803–812.

48. Lynch S, Ferrando SJ, Dornbush R, et al. Screening for brain fog: Is the montreal cognitive assessment an effective screening tool for neurocognitive complaints post-COVID-19? *Gen Hosp Psychiatry* 2022; 78: 80–86.

49. Manganotti P, Michelutti M, Furlanis G, et al. Deficient GABABergic and glutamatergic excitability in the motor cortex of patients with long-COVID and cognitive impairment. *Clin Neurophysiol* 2023; 151: 83–91.

50. McGregor G, Sandhu H, Bruce J, et al. Clinical effectiveness of an online supervised group physical and mental health rehabilitation

programme for adults with post-covid-19 condition (REGAIN study): multicentre randomised controlled trial. *BMJ* 2024; 384: e076506.

51. Writing Committee for the COMEBAC Study Group, Morin L, Savale L, et al. Four-Month Clinical Status of a Cohort of Patients After Hospitalization for COVID-19. *JAMA* 2021; 325: 1525–1534.

52. Nielsen TB, Leth S, Pedersen M, et al. Mental Fatigue, Activities of Daily Living, Sick Leave and Functional Status among Patients with Long COVID: A Cross-Sectional Study. *Int J Environ Res Public Health*; 19. Epub ahead of print 9 November 2022. DOI: 10.3390/ijerph192214739.

53. Niemczak CE, Ford JC, Roth RM, et al. Neuroimaging markers of cognitive fatigue in individuals with post-acute sequelae of SARS-CoV-2 infection. *Brain Cogn* 2025; 183: 106254.

54. Orfei MD, Porcari DE, D'Arcangelo S, et al. A New Look on Long-COVID Effects: The Functional Brain Fog Syndrome. *J Clin Med*; 11. Epub ahead of print 21 September 2022. DOI: 10.3390/jcm11195529.

55. Ortelli P, Quercia A, Cerasa A, et al. Lowered Delta Activity in Post-COVID-19 Patients with Fatigue and Cognitive Impairment. *Biomedicines*; 11. Epub ahead of print 8 August 2023. DOI: 10.3390/biomedicines11082228.

56. Ortelli P, Ferrazzoli D, Sebastianelli L, et al. Altered motor cortex physiology and dysexecutive syndrome in patients with fatigue and cognitive difficulties after mild COVID-19. *Eur J Neurol* 2022; 29: 1652–1662.

57. Rabaiotti P, Ciracì C, Donelli D, et al. Effects of Multidisciplinary Rehabilitation Enhanced with Neuropsychological Treatment on Post-Acute SARS-CoV-2 Cognitive Impairment (Brain Fog): An Observational Study. *Brain Sci*; 13. Epub ahead of print 12 May 2023. DOI: 10.3390/brainsci13050791.

58. Rossato MS, Brilli E, Ferri N, et al. Observational study on the benefit of a nutritional supplement, supporting immune function and energy metabolism, on chronic fatigue associated with the SARS-CoV-2 post-infection progress. *Clin Nutr ESPEN* 2021; 46: 510–518.

59. Ruzicka M, Sachenbacher S, Heimkes F, et al. Characterization of cognitive symptoms in post COVID-19 patients. *Eur Arch Psychiatry Clin Neurosci* 2024; 274: 1923–1934.

60. Taşkıran Sağ A. COVID-19 associated brain fog and neurocognitive assessment. *Cyprus J Med Sci* 2023; 8: 115–120.

61. Sathyamoorthy M, Sevak RJ, Cabrera J, et al. Enhanced External Counterpulsation Improves Cognitive Function of Persons With Long COVID. *Am J Phys Med Rehabil* 2024; 103: 734–739.

62. Schild A-K, Goereci Y, Scharfenberg D, et al. Multidomain cognitive impairment in non-hospitalized patients with the post-COVID-19

syndrome: results from a prospective monocentric cohort. *J Neurol* 2023; 270: 1215–1223.

63. Victoria LW, Oberlin LE, Ilieva IP, et al. A digital intervention for cognitive deficits following COVID-19: a randomized clinical trial. *Neuropsychopharmacology* 2024; 50: 472–479.

64. Whiteside DM, Basso MR, Naini SM, et al. Outcomes in post-acute sequelae of COVID-19 (PASC) at 6 months post-infection Part 1: Cognitive functioning. *Clin Neuropsychol* 2022; 36: 806–828.

65. Wright TJ, Sheffield-Moore M, Pyles RB, et al. Growth hormone treatment for neurologic symptoms of post-acute sequelae of COVID-19. *Clin Transl Sci* 2024; 17: e13826.
